# Building Scaffolds for Tubular Tissue Engineering

**DOI:** 10.3389/fbioe.2020.589960

**Published:** 2020-12-10

**Authors:** Alexander J. Boys, Sarah L. Barron, Damyan Tilev, Roisin M. Owens

**Affiliations:** Department of Chemical Engineering and Biotechnology, University of Cambridge, Cambridge, United Kingdom

**Keywords:** biomaterials, 3D printing, electrospinning, decellularization, lumen, vascular, intestine, trachea

## Abstract

Hollow organs and tissue systems drive various functions in the body. Many of these hollow or tubular systems, such as vasculature, the intestines, and the trachea, are common targets for tissue engineering, given their relevance to numerous diseases and body functions. As the field of tissue engineering has developed, numerous benchtop models have been produced as platforms for basic science and drug testing. Production of tubular scaffolds for different tissue engineering applications possesses many commonalities, such as the necessity for producing an intact tubular opening and for formation of semi-permeable epithelia or endothelia. As such, the field has converged on a series of manufacturing techniques for producing these structures. In this review, we discuss some of the most common tissue engineered applications within the context of tubular tissues and the methods by which these structures can be produced. We provide an overview of the general structure and anatomy for these tissue systems along with a series of general design criteria for tubular tissue engineering. We categorize methods for manufacturing tubular scaffolds as follows: casting, electrospinning, rolling, 3D printing, and decellularization. We discuss state-of-the-art models within the context of vascular, intestinal, and tracheal tissue engineering. Finally, we conclude with a discussion of the future for these fields.

## Introduction

Function of the human body is dependent on tubular tissues and tissue structures. These tissues, including vasculature, the intestines, the trachea, and many others, serve various roles in the body, ranging from absorption of nutrients to transport of oxygen. As may be expected given the broad assortment of functions associated with tubular tissues, these structures are susceptible to a variety of diseases and traumas. As such, significant focus has been placed on the generation of models of tubular systems for studies in disease, basic science, and drug discovery/efficacy. Many of these models utilize tissue engineering principles to recreate the function of these systems on the benchtop without requiring use of animal models (Bitar and Raghavan, 2012; Seifu et al., 2013; Law et al., 2016). The methods used to manufacture these tissue engineered systems play a major role in their resultant function. Here, we review the construction of tissue engineered systems for generating tubular models.

Tubular tissues have many unifying structural characteristics despite their various functions. Generally, these tissues are constructed in a lamellar manner, with sequential layers of tissue surrounding an internal opening. This opening, called the lumen, is where transport and containment of the specific medium for a particular tubular tissue occurs. This lumen is lined with a set of barrier-forming cells, called an epithelium (or endothelium in the case of vasculature). This structure functions to separate the internal contents of the lumen from the surrounding tissues and organs, while allowing selective permeation and transport across the epithelium. The epithelium is situated on a bed of extracellular matrix (ECM), which provides structural support for the lumen and the epithelium (Hendow et al., 2016). This ECM layer can be present in various forms, but it generally consists of cells embedded in connective tissue, including various proteins like collagen, elastin, etc. Depending on the function of the particular tissue, other tissue layers may also be present. For example, given the role of vasculature in moving blood throughout the body, blood vessels often contain a layer of smooth muscle, which assists in the vasodilation and constriction in the vascular system. Regardless of the particular function for a tubular tissue, the main purpose of these structures involves the separation of one media from another, guiding and transporting various fluids, gases, and solids.

Tissue engineering is the combination of cells and a template to generate a structure that recapitulates the native function of a specific tissue or tissue system. Often the template is a scaffold or hydrogel on which the cells can proliferate and produce ECM. Here, we discuss scaffolds and hydrogels nearly synonymously, as the manufacturing methods for producing a tubular scaffold versus a tubular hydrogel do not necessarily differ. However, these structures possess different fundamental properties, and each should be considered independently for a given application. An understanding of the native function and physiology of a tissue is often sufficient to inform the choice of cells and scaffold materials for a tissue engineered application. However, scaffold design is challenging, given the need to create a supportive structure for cells to grow and create the desired tissue, without impinging on the overall function of the resultant structure. These challenges are especially prominent in the design of tubular systems, given the need to create an intact lumen that can support the formation of an epithelium and other components. Various manufacturing approaches have been utilized for production of scaffolds, including casting, electrospinning, rolling, three-dimensional (3D) printing, and decellularization.

Determination of the resultant properties in tubular systems can be difficult, particularly due to geometry. Besides the manufacture of a tubular scaffold, characterization of the interaction between cellular and scaffold components, as well as their combined structural integrity, is fundamental. For example, the formation and continued integrity of the epithelial or endothelial barrier ensures the selective permeability of essential nutrients and metabolic by-products, while preventing the entrance of noxious or pathogenic compounds. Thus, without successful barrier formation, and characterization of such, the functionality and even survival of these models would be limited. Equally, any drug transport studies would be rendered invalid if barrier formation was insufficient. Intercellular junctions, provide this barrier function and consist of various proteins, such as cadherins, zonulin-1 (ZO-1), etc. Generally, membranes like the intestinal epithelium possess tight junctions, which highly regulate ionic/molecular passage across the epithelium, whereas the vascular endothelium, for example, is more permeable.

In this review, we discuss techniques commonly used to generate tissue engineered scaffolds for tubular systems. Our aim is to provide a categorization of the available methodologies for scaffold production to assist tissue engineers in navigating this extensive field. As such, we have chosen to focus on three specific tissue systems that represent various design challenges in the field: vasculature, the intestine, and the trachea for recapitulating the functions of many tubular tissue systems present in the body. Initially, we discuss the anatomy of these systems and some of the general criteria for tubular scaffold design. Then, we categorize the available scaffold manufacturing techniques. We proceed to examine some the applications of these techniques for our chosen tissue systems. Finally, we conclude with a discussion of the future for tissue engineered scaffold design for tubular tissue systems.

### Structure of Native Tubular Tissues

Various tubular tissues are present in the body, including the vascular system, digestive system, respiratory system, lymphatic system, reproductive system, and many others. As discussed above, we have chosen to highlight vasculature, intestines, and the trachea, as these applications are some of the most widely researched in terms of generation of tubular tissue engineered models (Bitar and Raghavan, 2012; Seifu et al., 2013; Hendow et al., 2016; Law et al., 2016). Additionally, these systems possess various functions that differentiate them from one another with respect to design. Below, we discuss the specifics of the structure and physiology for each of these systems.

#### Vascular Structure

**FIGURE 1:**
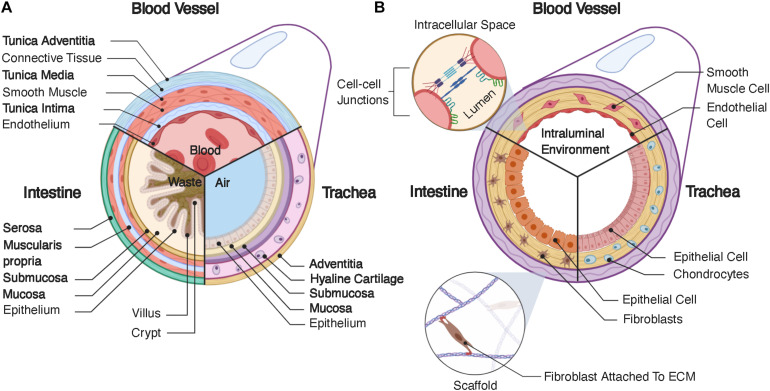
**(A)** Structure of native tubular tissue systems. Specifically, this schematic highlights the structure of vasculature, the intestines, and the trachea. **(B)** Typical structure for tissue engineered tubular systems corresponding the native systems in **(A)**.

The primary role of the vascular system is the transport of blood throughout the body at relatively high velocities (Riva et al., 1985; Klarhöfer et al., 2001), generating significant fluid shear stress on the walls of a blood vessel (Akintewe et al., 2017). Additionally, the vascular endothelium is relatively permeable, allowing transport of biochemical factors through the vascular wall and even cells during some disease states (Park-Windhol and D’Amore, 2016). Blood vessels range in size from capillaries and microvasculature, which are only microns in diameter (Sieminski and Gooch, 2000), to larger veins and arteries, which can be ∼30mm in diameter in the case of the pulmonary artery (Kuriyama et al., 1984). Small capillaries, such as those that make up the blood brain barrier, can consist of only one cell, wrapped onto itself to create the interior lumen (Abbott et al., 2006). However, we will focus on larger blood vessels that have a lamellar structure divided into three layers (Figure 1A): the tunica intima, tunica media, and tunica adventitia. Generally, the intima contains the endothelium, the media is composed of a layer of smooth muscle, and the adventitia consists of a layer of connective tissue (James and Allen, 2018). The endothelial layer of cells makes up the vascular wall. These cells form a semi-permeable membrane that allows transport of nutrients, oxygenation, and waste removal from surrounding tissues (Park-Windhol and D’Amore, 2016). The layer of smooth muscle in the tunica media aids in control of vasodilation, which can regulate local blood flow. Lastly, the tunica adventitia provides support for the internal layers in addition to housing a variety of nerves, immune cells, and other support systems for vasculature (James and Allen, 2018). The heart pumps blood through the luminal compartment of these vessels. This pumping creates relatively high rates of fluid flow, ∼30 mL/min (Klarhöfer et al., 2001), thereby generating significant fluid shear on the walls of vasculature, which is an additional necessary consideration in any tissue engineered model.

#### Intestinal Structure

The intestines are a portion of the gastrointestinal tract, which extends from the mouth, through the esophagus, the stomach, the small intestines, the large intestines, and finally to the rectum and anus. The main function of the intestine is to absorb nutrients from food and liquids we ingest and expel the remaining waste out of the body, with each portion of the gastrointestinal tract consisting of four layers. Starting from the interior lining of the lumen, these layers are the mucosa, submucosa, muscularis propria, and serosa (Figure 1A). In this review, we focus on the intestines and describe the layers in the context of these organs, versus other portions of the gastrointestinal tract. The mucosa contains the intestinal epithelium and has absorptive, secretory, and protective functions. The intestinal epithelium is a tight barrier system, robustly separating the interior contents of the lumen from the surrounding tissue (Suzuki, 2013). Intestinal epithelial tissue has a complex 3D structure, consistent of luminal projections, called villi, with intermediate invaginations, called crypts (Santos et al., 2018). This 3D architecture maximizes interior surface area, aiding in nutrient adsorption (Rao and Wang, 2010). The intestinal epithelium consists of numerous cell types with various functions, which have a semi-regimented distribution along this 3D structure. Generally, these cells and their respective functions are as follows: enterocytes – absorption of nutrients and formation of intestinal barrier; goblet cells – secretion of mucin; enteroendocrine cells – sensing of nutrients and microbes and communication with the enteric nervous system; transit amplifying cells – differentiation toward secretory or absorptive lineages; as well as tuft cells, Paneth cells, intestinal stem cells, and others (Santos et al., 2018). These epithelial cells are adhered to the lamina propria, a layer of connective tissue, which is surrounded by a sheet of smooth muscle cells. The next layer is the submucosa, which contains a series of immune cells, nerves, and lymphatic cells. This layer is surrounded by the muscularis propria, which provides peristaltic pumping through muscle cells, performing the critical function of gut motility. Finally, the outermost layer is the serosa, or in some cases the adventitia depending on the present populations of cells, which forms a barrier around the gastrointestinal tract (Rao and Wang, 2010). The gut also possesses a complex series of nerves called the enteric nervous system, which consist of two parallel nerve plexi, the submucosal plexus and myenteric plexus, which run along the length of the gastrointestinal tract (Furness, 2012). Lastly, the interior of the gut contains a large cohort of bacteria, called the gut microbiome, which can influence various other organs throughout the body (Cryan et al., 2019), in addition to further complicating tissue engineered design.

#### Tracheal Structure

The trachea is fundamental in swallowing, speech and respiratory processes. It resides below the upper airways (nasal cavity, larynx, pharynx) and forms part of the lower airways (trachea, bronchi, bronchioles, alveoli), with its main function to conduct and warm air (Brand-Saberi and Schäfer, 2014). The trachea is comprised of four main layers (Figure 1A)**:** mucosa, submucosa, hyaline cartilage, and adventitia. The mucosa contains a pseudostratified epithelium, which lines the lumen and contains many cell types including secretory club cells, ciliated cells, mucus producing goblet cells, basal stem cells, and pulmonary neuroendocrine cells. Ciliated, mucus-producing, and secretory cells act in coordination to aid mucociliary clearance and protection against infection, while basal cells aid in regenerative processes (Brand-Saberi and Schäfer, 2014). The submucosa is a connective tissue layer containing submucosal glands, which contribute to mucus secretion. The cartilage layer consists of horseshoe-like rings of hyaline cartilage joined by fibroelastic tissue, which, is closed posteriorly by a membranous structure consisting of longitudinally oriented smooth muscle. Lastly, the adventitia consists of connective tissue. Both the cartilage and adventitial layers are fundamental in producing the unique structural and mechanical properties of the trachea. For example, the specific flexibility which permits the rotation and flexion of the neck while also maintaining sufficient structural strength to withstand compression and pressure alterations during respiratory processes. In order to reflect and integrate with the *in vivo* environment, tracheal tissue engineered models must adhere to these mechanical requirements (Boazak and Auguste, 2018). The adventitia also houses numerous other cell types, such as fibroblasts, adipocytes, nerves, and connections to vasculature, which is essential in meeting blood, nutrient, and metabolic demands. The respiratory system requires an air-liquid interface between the interior of the lumen and the surrounding epithelium (Pezzulo et al., 2011; de Souza Carvalho et al., 2014), creating a different environment from the fluidic environments of the vascular and intestinal systems, which can be difficult to produce in tissue engineered models. Unlike the *in vitro* models discussed for vasculature and the intestine, the outlook for tracheal tissue engineering so far largely concerns implantable scaffolds for tracheal replacement (Bogan et al., 2016; Law et al., 2016; Etienne et al., 2018). Indeed, the trachea is subject to a range of airway disorders which may result from infection, stenosis, collapse, or cancer (Etienne et al., 2018). The rise of biomedical engineering approaches, which recapitulate tracheal tissue, have been largely motivated by these applications. Here, we will discuss some of these studies, which focus on implant generation, and how they can be further developed for use as benchtop disease models.

### Design Criteria for Tissue Engineering Tubular Systems

The physiology of tubular tissues is often complex, requiring various factors to produce an approximate model of the desired tissue. Generally, tissue engineered systems utilize a cell type (or types) in combination with a scaffold to recreate the primary function (or functions) of the tissue. However, choosing appropriate cell types and scaffold architectures can be difficult. Here, we have highlighted some of the necessary design criteria to consider for manufacturing a scaffold for tubular tissue engineering (Table 1).

**TABLE 1:** Design criteria for tubular tissue engineered scaffold development as a function of tissue type.

Tissue System	Cellular				Mechanical			Other	
	Contiguous Epithelium/Endothelium	Smooth Muscle Layer	Supporting Connective Tissue Layer	Mucous Layer	Fluid Shear	Pressurization	Mechanical Stimulation (Peristalsis)	Separation of Luminal Chamber	Air-liquid Interface
Vasculature	X	X	X		X	X			
Intestine	X	X	X	X	X	X	X	X	
Trachea	X		X	X		X		X	X

Scaffold design for tubular systems presents a variety of challenges. First, one must consider the source and types of cells, with any tubular system requiring a source of epithelial cells. However, the exact behavior of these cells will vary depending on the tissue in question. Epithelial cells are often co-cultured with ECM-producing cells like fibroblasts or smooth muscle cells (Boland et al., 2004; Yoshikawa et al., 2011; Chen et al., 2015), but even the type of matrix-producing cell can vary depending on application. For example, the trachea requires production of cartilaginous ECM using chondrocytes (Lin et al., 2009) or mesenchymal stem cells differentiated along chondrogenic pathways (Asnaghi et al., 2009; Haykal et al., 2014). Different tissues will also require different supporting cells. For example, native intestinal epithelium contains goblet cells for producing mucus (Dosh et al., 2019). Both native vasculature and intestine possess a layer of musculature (Boland et al., 2004), necessitating sourcing of appropriate muscle cells. We have summarized some common cell lines or primary cells used to reconstitute native function in tissue engineered models (Table 2).

**TABLE 2:** Commonly used cells for tissue engineered models of vasculature, the intestine, and the trachea.

Tissue System	Native Tissue Layer	Cell Model	Cell Function	References
Blood Vessel	Tunica Intima	Human Umbilical Vein Endothelial Cell (HUVEC)	Endothelial Cell	Boland et al., 2004; Lovett et al., 2007; Du et al., 2012; Wang et al., 2014; Cui et al., 2019
		Endothelial Progenitor Cell (EPC)	Endothelial Cell	Neff et al., 2011; Ju et al., 2017; Atchison et al., 2017
		Primary Endothelial Cell	Endothelial Cell	Matsuda, 2004, 200; Opitz et al., 2004; Zang et al., 2013
	Tunica Media	Primary Smooth Muscle Cells	Smooth Muscle Cell	Seliktar et al., 2003; Opitz et al., 2004; Swartz et al., 2005; Lee et al., 2007; Zhang et al., 2013; Fu et al., 2014; Cui et al., 2019
	Tunica Adventitia	Dermal Fibroblasts	Fibroblast	Seliktar et al., 2003; Boland et al., 2004
Intestine	Mucosa	Caco-2 Cells	Enterocyte	Costello et al., 2014; Chen et al., 2015; Ladd et al., 2018
		HT-29-MTX Cells	Goblet Cell	Chen et al., 2015
	Submucosa	Primary Intestinal Myofibroblast	Myofibroblast	Chen et al., 2015
	Muscularis Propria	Smooth Muscle Cell	Smooth Muscle Cell	Zakhem et al., 2012; Knight et al., 2013
	Serosa			
Trachea	Mucosa	Primary Respiratory Epithelial Cell	Epithelial Cell	Butler et al., 2017; Kreimendahl et al., 2019; Park et al., 2019
		Turbinate Mesenchymal Stromal Cell	Epithelial Cell	Park et al., 2018; Ahn et al., 2019
	Submucosa			
	Hyaline Cartilage	Mesenchymal Stem Cell	Chondrocyte	Wang et al., 2020
		Adipose-derived Stem Cell	Chondrocyte	Giraldo-Gomez et al., 2019
		Auricular Chondrocyte	Chondrocyte	Park et al., 2019
	Adventitia	Nasal Fibroblast	Fibroblast	Kreimendahl et al., 2019

A major requirement for every tubular scaffold is the formation of a contiguous epithelial or endothelial lining (Figure 1B). Cells are most effectively seeded homogenously on two-dimensional (2D), non-porous surfaces, such as cell culture flasks, or in injectable media, such as hydrogels. However, tubular scaffolds are not flat and, generally, are porous. Therefore, the necessity for homogenous seeding on a 3D surface, requires alternative methodologies. For example, researchers have seeded cells onto flat membranes and then rolled these membranes into tubes (Yuan et al., 2012; Cheng et al., 2017; Zhao et al., 2018). Other studies have used dynamic methods, relying on the cells to adhere homogenously to the surrounding walls through rotational or pressurized actuation (Niklason and Langer, 1997; Godbey et al., 2004; Nieponice et al., 2008). Porous scaffolds are beneficial in that they provide greater access to media by cells, but these pores also make the formation of a contiguous epithelium difficult. Some studies have back-filled pores with ECM-producing cells or depositing cells in a layered approach to assist in the formation of an epithelium (Liu et al., 2015; Chen et al., 2015). Many implant-driven studies also rely on cell infiltration *in vivo*. All of these methods have limitations, but continuous iteration has improved the feasibility of accomplishing this particular task for tubular tissue engineering.

Another necessary design consideration is provision of nutrients to all cells in the system. Tubular systems will inherently contain epithelial cells, but they may also contain supporting cells, present underneath the epithelium (Figure 1B). In the case of vascular systems, these underlying cells can theoretically receive nutrients through the endothelial wall, as the vascular endothelium is inherently permeable for this purpose. However, intestinal and respiratory tissue engineering presents a particular challenge, as the lumen should not be used to provide nutrients to any of the cells in the system, given the function of these tissues (Bitar and Raghavan, 2012). In the body, nutrients are provided to the tissues underlying the epithelium by surrounding vasculature. To mimic native tissues, tissue engineered models rely on perfusion of the scaffold with media to simulate nutrient transport. However, separation of the luminal compartment from the surrounding scaffold is difficult. Custom bioreactors can accomplish this task (Haykal et al., 2014; Zhou et al., 2018), but bioreactor design and setup also complicate culture for the system.

**FIGURE 2:**
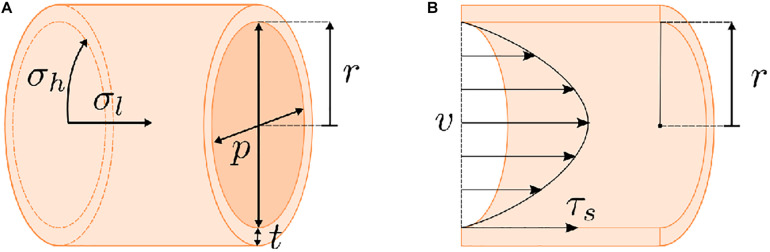
Stresses present for dynamic flow of a fluid through a tube. **(A)** Pumping a liquid or gaseous medium through a tube will generate a pressure on the tube walls. The highest stress resultant from this pressurization is the hoop stress (σ_h_). Any scaffold must possess sufficient strength to compensate for this hoop stress. **(B)** Fluid movement will also result in a shear stress (τ_s_) on the walls of the tube. Generally, these factors (σ_h_ and τ_s_) can be calculated based on the geometry of the tube, i.e., wall thickness (t) and inner radius (r), the pressure (p) on the tube walls, the flow rate of the fluid (v), and the viscosity of the fluid. However, these calculations are complicated by scaffold porosity and the potential for effects of cell growth on the scaffold over the course of an experiment.

Secondary design criteria relate to the specific function of the tubular system. For example, vascular systems often induce fluid movement to mimic blood flow. As such, vascular systems utilize external pumps, typically with pulsatile pumping patterns (Niklason and Langer, 1997; Niklason et al., 1999; Opitz et al., 2004), which also necessitates the ability of the scaffold to withstand mechanical forces that result from pumping, i.e. pressurization and fluid shear (Figure 2). Fluid shear is also known to drive vascular endothelial phenotype and morphology (Wang et al., 2013; Wang et al., 2016a; Polacheck et al., 2017) and regulates angiogenesis *in vitro* (Song and Munn, 2011; Galie et al., 2014), which can be beneficial in different tissue engineering models. An accurate intestinal model requires peristaltic pumping to mimic gut motility. Peristalsis has been achieved using external bioreactors (Zhou et al., 2018), but bioreactors are often custom-made, requiring further design and optimization. Secondarily, native intestinal environments contain a bacterial cohort. Inputting of bacteria into a tissue engineered lumen is feasible (Costello et al., 2014) but also further complicates culture conditions. Other systems, like the trachea, require the presence of air in the lumen, which can make culture condition more complex. Many studies have utilized bioreactors that rotate the tubular tracheal scaffold along its axis with half of the scaffold submerged in media and the other half in air to create an air-liquid interface (Lin et al., 2009). However, this approach is not ideal for biomimetic studies assessing drug delivery, given its dissimilarities to the native tracheal environment.

## Methods for Fabricating Tubular Scaffolds

Various methodologies can be used to produce tubular scaffolds for tissue engineering. We have divided these techniques into five categories (Table 3) to assist in experimental design and planning. Below, we have discussed the general methodologies for each technique, while also addressing their benefits and limitations. Other reviews have also examined general strategies for tissue engineering tubular systems (Bitar and Raghavan, 2012; Seifu et al., 2013; Hendow et al., 2016; Law et al., 2016; Song et al., 2018).

**TABLE 3:** Categorization of techniques for manufacturing tubular scaffolds for tissue engineering.

Fabrication method	Advantages	Disadvantages	References
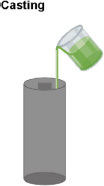	• Compatible with most material types • Simple and easy to implement • Applicable with cell-seeded materials	• Difficult to produce complex shapes • Processing can result in toxic byproducts • Secondary cell seeding method required	Vasculature Seliktar et al., 2003; Matsuda, 2004; Opitz et al., 2004; Swartz et al., 2005; Liu et al., 2007; Nieponice et al., 2008; Ma et al., 2010; Wang et al., 2014; Guo et al., 2017; Atchison et al., 2017; Strobel et al., 2018b; Im et al., 2019 Intestine Yu et al., 2012; Zakhem et al., 2012; Chen et al., 2015; Zhou et al., 2018; Ladd et al., 2018, 2019; Roh et al., 2019 Trachea Naito et al., 2011
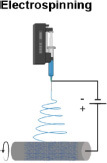	• High degree of control over scaffold properties (porosity, mechanics, etc.) • Easily applied for tube formation • Directly compatible with proteins	• Processing can result in toxic byproducts • Secondary cell seeding method required • Optimization necessary for experimental setup	Vascular Boland et al., 2004; Lee et al., 2007; Smith et al., 2008; Wang et al., 2009; Han et al., 2011; Du et al., 2012; Zhang et al., 2013; Fu et al., 2014; Zhou et al., 2016; Ju et al., 2017; Strobel et al., 2018a; Rodriguez et al., 2019 Intestine Yoon and Kim, 2010; Knight et al., 2013 Trachea Hinderer et al., 2012; Mahoney et al., 2016; Wu et al., 2017; Best et al., 2018; Kang et al., 2019; O’Leary et al., 2020
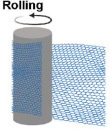	• Cells can be seeded in 2D and rolled into 3D tube • Simple and easy to implement	• Production may require handling of cell-seeded scaffold • Sealing of tube can be difficult	Vascular Niklason et al., 2001; Shen et al., 2003; L’Heureux et al., 2006; Pricci et al., 2009; Konig et al., 2009; Gauvin et al., 2010; Rayatpisheh et al., 2014; Jung et al., 2015; Gui et al., 2016; Zhao et al., 2018; Wang et al., 2018 Intestine Grikscheit et al., 2002, 2004
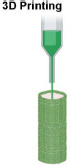	• High degree of customization and control over scaffold production • Compatible with most material types • Applicable with cell-seeded materials	• Expensive equipment requires for production of scaffolds • Optimization necessary for experimental setup • Some printing techniques do not currently possess high resolution	Vascular Melchiorri et al., 2016; Rabionet et al., 2018; Cui et al., 2019 Trachea Johnson et al., 2016; Gao et al., 2017; Taniguchi et al., 2018; Hsieh et al., 2018; Park et al., 2018; Park et al., 2019; Machino et al., 2019; Xia et al., 2019; Kang et al., 2019; Ahn et al., 2019; Gao et al., 2019
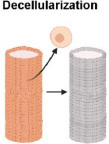	• Scaffold material is highly biocompatible • Intrinsic biochemical factors can benefit production of tissue engineered model	• Secondary cell seeding method required • Decellularized scaffold can contain biochemical factors that negatively affect production of tissue engineered model • Extensive characterization and quality control are necessary	Vascular McFetridge et al., 2007; Xi-Xun et al., 2008; Yang et al., 2009; Neff et al., 2011; Lee et al., 2012 Intestine Totonelli et al., 2012 Trachea Johnson et al., 2016; Butler et al., 2017; Ghorbani et al., 2017; Zhong et al., 2019; Batioglu-Karaaltin et al., 2019; Giraldo-Gomez et al., 2019; Wang et al., 2020

### Casting

Casting is one of the most commonly used manufacturing techniques across the entirety of the tissue engineering field. The basis for this methodology is the pouring or injection of a liquid into a mold, at which point the liquid is induced to form a solid structure. The liquid can be derived from a variety of means, including the melting and solidification of a material (Im et al., 2019), the solubilization of a material in a solvent and subsequent evaporation of the solvent (Mooney et al., 1995; Opitz et al., 2004; Nieponice et al., 2008; Ma et al., 2010; Zakhem et al., 2012), or the cross-linking (Matsuda, 2004; Guo et al., 2017) or gelation of a material into a solid or semi-solid structure, such as a hydrogel (Wang et al., 2014; Strobel et al., 2018b). Casting is also applicable within the context of more complex molding techniques, such as vacuum-assisted tube formation (Singh et al., 2017), and for the formation of more complex structures (Ladd et al., 2018). As may be expected, the chosen material will dictate the mechanism. In the case of some hydrogels, particularly biomolecular gels, e.g., collagen, fibrin, etc., cells can be cast with the gel (Seliktar et al., 2003; Swartz et al., 2005; Liu et al., 2007; Naito et al., 2011; Atchison et al., 2017, 2020). This method is convenient and widely applicable, as it can be used to produce a variety of geometries and shapes with homogenous cell populations and is compatible with other manufacturing methods (Atchison et al., 2017; Iannucci et al., 2019). However, a secondary seeding step is often still necessary to create the stratified cellular structure of native tubular tissues, even when using homogeneously seeded hydrogels.

In tissue engineering cases, the inclusion of pores in the final structure is often necessary to provide nutrient access for seeded cells. Pores can be created in variety of manners but are often produced using a secondary material or porogen that can be removed through post-processing, leaving a pore in its place. Various porous scaffolds have been produced using ice templating (Boccaccini et al., 2005; Shin’oka et al., 2005; Nieponice et al., 2008; Ma et al., 2010; Zakhem et al., 2012; Chen et al., 2015; Roh et al., 2019). This process involves freezing a solution or mixture of water and the scaffold material, followed by sublimation of the ice, also called lyophilization, leaving pores in the resultant solid structure. Alternatively, pores can be produced by including orthogonally soluble solids in the casting solution. For example, salt can be included in solvent-cast polymeric solutions and then washed out with water after solvent evaporation (Mooney et al., 1995; Sin et al., 2010; Costello et al., 2014). This technique is only feasible for highly porous materials, because lower porosities will prevent access for porogen removal.

**FIGURE 3:**
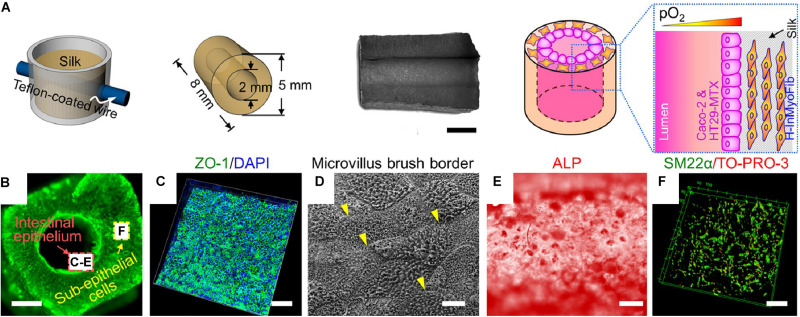
Example of tubular scaffold for intestinal tissue engineering constructed through the casting process (Chen et al., 2015). **(A)** Schematic showing scaffold production. Silk is lyophilized in a mold to create a porous scaffold with a central lumen. Cells are seeded into the scaffold and into the interior of the lumen. **(B)** Image showing scaffold lumen. Scale bar is 4 mm. **(C)** Immunostain for ZO-1 and cell nuclei (DAPI). Scale bar is 100 μm. **(D)** Scanning electron microscopic (SEM) image of epithelial lining. Scale bar is 1 μm. **(E)** Alkaline phosphatase (ALP) staining for ALP enzymatic activity on interior lining of lumen. Scale bar is 200 μm. **(F)** Confocal z-stack of cells in scaffold immunostained for SM22a, a marker for myofibroblasts. Scale bar is 50 μm. Images were reordered from multiple panels, and lettering has been relabeled for consistency as part of this review article (Chen et al., 2015). These images are reprinted under Creative Commons Attribution 4.0 International License, available at http://creativecommons.org/licenses/by/4.0/.

Casting is simple, convenient, and compatible with a wide range of materials, with examples showing the application of casting techniques for the production of complex models with stratified layers of cells (Figure 3). However, various factors can complicate the casting process, including the construction of larger objects, objects with inconsistent cross-sections, or objects with internal cavities. In these cases, mold design is particularly important. Inclusion of vents can ensure filling of the entire mold. However, releasing the resultant material from a mold can also be difficult, depending on the material. Regardless, complex shapes can be cast to generate useful, tissue-like structures (Wang et al., 2014). Another negative factor affecting casts, particularly solvent cast polymers and some porogen forming techniques, is the presence of remnant solvent or toxic porogens that can negatively affect cell growth. These issues can be avoided with proper handling and preparation of the cast. Seeding cells into cast tubes can also be difficult, as discussed above. Many studies seed cells into the lumen through pipetting. However, the scaffolds often need to be rotated (Opitz et al., 2004; Atchison et al., 2017, 2020) or subjected to another form of dynamic seeding (Godbey et al., 2004; Nieponice et al., 2008) to reach homogeneous seeding on the interior lining of the scaffold. Nevertheless, casting remains a commonly used mechanism for tubular scaffold production given its customizability and wide range of accessible materials. Further, the resultant tubular scaffolds are repeatably manufacturable, requiring no further steps for assembly after the initial cast. These factors make this technique widely applicable in the field of tubular tissue engineering and beyond.

### Electrospinning

**FIGURE 4:**
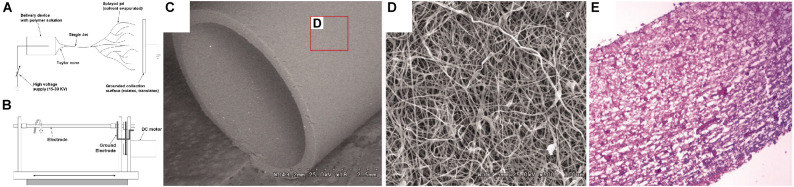
Example of electrospinning process for manufacturing a tubular scaffold for vascular tissue engineering (Lee et al., 2007). **(A)** Schematic of electrospinning process. **(B)** Schematic of spinning mandrel for tubular scaffold production. **(C)** SEM image of bulk scaffold, produced from collagen, elastin, and polymeric mixture, at 18x magnification. **(D)** SEM image of scaffold from inset in **(C)** at 500x magnification. **(E)** Hematoxylin and eosin histological stain of scaffold seeded with smooth muscle cells. Images were reordered from multiple panels, and lettering has been relabeled for consistency as part of this review article (Lee et al., 2007).

Electrospinning techniques involve the solubilization of a polymeric or biomolecular material, which is ejected from a syringe. During the electrospinning processes, the fluid is charged through an applied voltage and directed toward a neutral or oppositely charged mandrel. The solvent evaporates as the material travels toward the mandrel, creating nanofibers. For the formation of tubular scaffolds, the mandrel is typically rotated during the extrusion process. The resultant mesh of nanofibers can be removed from the mandrel, producing a mesh-tube (Rocco et al., 2014), with examples producing contiguous tubular structures that are compatible with cell-seeding (Figure 4). In many cases these meshes are combined with secondary electrospinning, deposition, or casting techniques to change the properties and/or structure of the scaffold. The process of electrospinning, particularly for scaffold production, has been covered extensively in previous reviews (Pham et al., 2006; Rocco et al., 2014).

Electrospinning provides a high degree of control over the resultant “pore” size of the mesh and the mechanical properties of the fibers. This process is also compatible with numerous materials that are suitable for scaffold production (Figure 4). Most electrospun scaffolds have been produced using polymers, or polymers mixed with ECM proteins (Vaz et al., 2005; Buttafoco et al., 2006; Lee et al., 2007; Smith et al., 2008; Wang et al., 2009; Yoon and Kim, 2010, 201; Han et al., 2011; Du et al., 2012; Hinderer et al., 2012; Zhang et al., 2013; Knight et al., 2013; Fu et al., 2014; Ott et al., 2016; Mahoney et al., 2016; Zhou et al., 2016; Wu et al., 2017; Ju et al., 2017; Best et al., 2018; Strobel et al., 2018a; O’Leary et al., 2020). However, some scaffolds have purely utilized proteins (Boland et al., 2004). Electrospun scaffolds suffer from the same disadvantages as the cast scaffolds above, namely toxicity from remnant solvent and potential difficulties in homogeneous cell seeding. However, both of these criteria have been examined extensively. A similar technique to electrospinning, called gel spinning, has also been used for tubular scaffold production, where high viscosity gels are extruded onto a spinning mandrel (Lovett et al., 2008; Rodriguez et al., 2019). Generally, electrospinning and other similar techniques have been widely and successfully applied for the production of tubular scaffolds.

### Rolling

**FIGURE 5:**
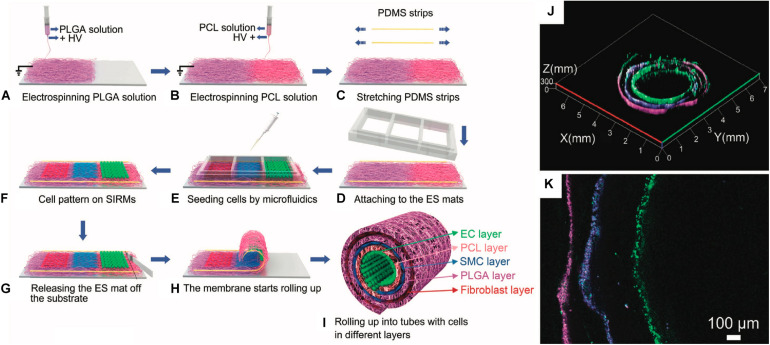
Example of rolling process for a vascular tissue engineered tubular construct (Cheng et al., 2017). **(A–I)** Schematic detailing process for producing cell-seeded, stress-induced rolling membrane (SIRM). **(A–C)** Poly(dimethyl siloxane) (PDMS) substrate is coated with poly(DL-lactide-co-glycolide) (PLGA) and poly(ε-caprolactone) (PCL) through electrospinning (ES) under high voltage (HV). **(D–F)** Resultant substrate is seeded with endothelial cells (ECs), smooth muscle cells (SMCs), and fibroblasts. **(G–I)** Scaffold is released from pre-stressed substrate, causing rolling. **(J,K)** Resultant stratified cell layers in rolled substrate: ECs are shown in green, SMCs are shown in blue, and fibroblasts are shown in magenta. Images were reordered from multiple panels, and lettering has been relabeled for consistency as part of this review article (Cheng et al., 2017).

Rolling mechanisms involve the rolling of a flat substrate into a tube. Rolling is usually accomplished using a mandrel to manually roll the substrate. However, some studies have generated tubes with stratified cell layers using a self-assembly mechanism based on properties of the underlying substrate (Figure 5). Some of the earlier studies to produce tubular scaffolds for tissue engineering used rolling methodologies. Generally, these studies would produce a polymeric sheet and stitch the sheet into a tube (Niklason and Langer, 1997; Niklason et al., 1999; Niklason et al., 2001; Gui et al., 2011; 2016). Other studies developed the use of cell-derived ECM sheets that were rolled into tubes using a mandrel (L’Heureux et al., 1998, 2006; Pricci et al., 2009; Konig et al., 2009; Gauvin et al., 2010; Jung et al., 2015). This approach was particularly interesting in its use of only biological materials. In these studies, ECM-producing cells were grown to confluence. The resulting ECM sheet was then detached and rolled into a tube, where further cells could be seeded. One study used an electrospun scaffold to assist in rolling a cell sheet into a tubular construct (Rayatpisheh et al., 2014). Other studies also focused on rolling polymeric sheets around a mandrel (Shen et al., 2003; Wang et al., 2016b, 2018). More recently, groups have developed self-assembling tubes. Self-assembly mechanisms or other rolling strategies that can be performed sterilely have the major benefit of allowing for cell seeding prior to rolling. Rolling is initiated in these studies using either mechanically tensioned sheets bound (Cheng et al., 2017) or shape-memory polymers (Zhao et al., 2018). In either of these scenarios, cells can be homogeneously seeded and cultured in 2D and then rolled into a 3D tube (Figure 5). This strategy allows for the effective production of a confluent monolayer of epithelial cells on a structure easily compatible with typical cell culture techniques, while still ultimately producing a tubular tissue engineered structure. However, once the structure has been rolled, the sealing of the tube from the free edges of the rolled substrate needs to be addressed. Many strategies simply use multi-layered tubes, but this approach can potentially limit media access to the basal side of the seeded cells. Alternatively, tubes can be closed with stitching, as described above, or through use of a sealant to seal the free edges of the tube (Grikscheit et al., 2002, 2004). Rolling is perhaps the only manufacturing method that most specifically applies to tubular scaffold production, and, as such, has had significant impact on this field.

### 3D Printing

**FIGURE 6:**
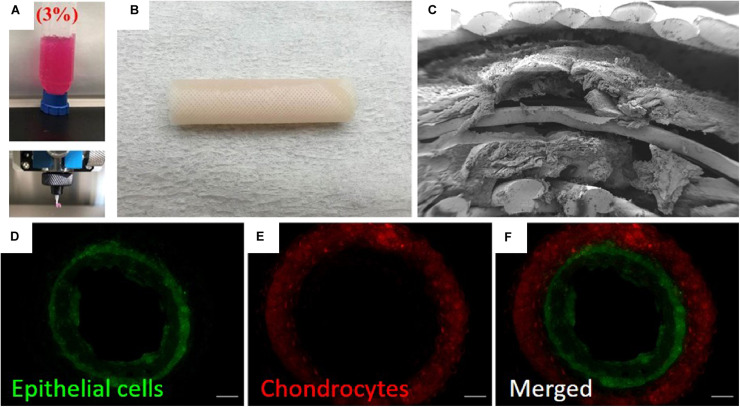
Example of 3D printing process for construction of tissue engineered trachea (Park et al., 2019). **(A)** Image showing 3D printing of alginate into a cell-compatible hydrogel. **(B)** Image of 3D printed trachea. Scaffold consisted of 5 layers (innermost to outermost): gridded pattern of poly(capralactone), alginate hydrogel containing primary nasal epithelial cells, cylindrical pattern of poly(capralactone), alginate hydrogel containing primary auricular chondrocytes, gridded pattern of poly(capralactone). **(C)** SEM image showing lamellar structure of scaffold. **(D–F)** Fluorescence microscopy images showing stratified cell layers in scaffold cross-section. Epithelial cells are shown in green, and chondrocytes are shown in red. Images were reordered from multiple panels, and lettering has been relabeled for consistency as part of this review article (Park et al., 2019). These images are reprinted under Creative Commons Attribution 4.0 International License, available at http://creativecommons.org/licenses/by/4.0/.

3D printing, also known as additive manufacturing or bioprinting in some tissue engineering cases, is the process of forming a 3D structure in a layer-by-layer manner. Printing processes typically involve extrusion of a material from a nozzle or photo-crosslinking of an object from a liquid precursor. In extrusion-based 3D printing, a liquid material, similar to those used for casting approaches, is extruded from a nozzle onto a platform. The nozzle follows a fabrication path across the platform, generating a single layer of the ultimate desired shape. Once this layer has set, the nozzle ejects a second layer of material on top of the initial layer, thereby constructing a 3D object (Zhu et al., 2016). This type of 3D printing is compatible with most material types, including hydrogels and hydrogels containing live cellular populations. One study used polymeric scaffolds and cell-seeded hydrogels to produce layered structures containing multiple cell populations for construction of a tissue engineered trachea (Figure 6). For bioprinting applications, cell-seeded hydrogels or other cell-compatible, printable materials are often called bioinks. However, specialized printers can be required depending on the material. Another format of 3D printing involves the photo-crosslinking of a polymer precursor from a liquid bath. In these printers, the precursor is cross-linked and fused to a baseplate, which is moved in 3D as subsequent layers are crosslinked onto the initial layer, generating 3D structures in this manner. This type of printing is typically performed using a photo-initiated cross-linker, meaning that material choices are limited to those which can be constructed as such. Inkjet printing is also frequently used in biological applications, but this type of printing is not typically compatible with the creation of large, 3D scaffolds like those used for tissue engineering and, as such, will not be discussed here. Other novel types of printing are also being developed, which will undoubtably apply to the production of tubular scaffolds. Various reviews have specifically focused on 3D printing for tissue engineering and the available methods (Wang et al., 2015; Zhu et al., 2016; Galliger et al., 2019).

A major advantage to the 3D printing process is the potential for easily customizable scaffolds and, when using compatible printers, the potential print materials containing live cells (Park et al., 2019; Cui et al., 2019). Unlike casting, this type of bioprinting is more easily compatible with the generation of stratified layers of cells. However, the resolution of bioprinters is often limited due to the viscous properties of biomolecular pre-gel solutions. Studies have directly examined the optimization of these properties to enhance bioprinting efforts (Diamantides et al., 2019). Excluding bioprinting, other 3D printed scaffolds suffer from the same drawbacks in cell seeding as cast or electrospun scaffolds but can still produce functional tubular scaffolds (Melchiorri et al., 2016; Gao et al., 2017; Rabionet et al., 2018; Hsieh et al., 2018; Park et al., 2018; Xia et al., 2019). 3D printing methodologies are also compatible with other techniques discussed in this review. For example, one study focused on the construction of a 3D printed scaffold, which was then molded with a cell-laden hydrogel through casting (Gao et al., 2019). Another study used a combination of 3D printing and electrospinning techniques (Ahn et al., 2019). Studies are now increasingly focusing on the use of 3D printing to create vascular structures (Abaci et al., 2016; Lei et al., 2019), which could potentially be used to provide nutrients to larger structures, such as intestinal models. Recently, some studies have printed a trachea directly using spheroid cell cultures (Taniguchi et al., 2018; Machino et al., 2019). These studies and others demonstrate the wide applicability and relevance of 3D printing in various areas of these fields.

### Decellularization

Decellularization involves the production of a scaffold from native tissue. This field encompasses a wide variety of applications, from whole organ decellularization (Gilbert et al., 2006; Crapo et al., 2011) to decellularization of specific tissues and engineering of these structures for other uses (Lee et al., 2012; Boys et al., 2019). Generally, decellularization proceeds through the explanation of a native tissue and then treatment for the complete removal of cells from the tissue. For cell removal, the tissue is typically subjected to a series of washes with enzymes or detergents to lyse and remove the native population of cells. After removal, the resultant tissues are washed further or lyophilized to prepare the structure as a scaffold for tissue engineering (Gilbert et al., 2006). In tissue engineering applications, decellularized scaffolds may be used for allograft transplantation or implantation. However, the scaffold must also be re-seeded with an appropriate cellular population to recapitulate the desired tissue system *in vitro* (McFetridge et al., 2007; Xi-Xun et al., 2008; Yang et al., 2009; Neff et al., 2011; Zang et al., 2013; Bertanha et al., 2014). Recellularization is often accomplished through perfusion of the scaffold with a new cellular population. Decellularization methods are not reserved exclusively for mammalian tissues. Some studies have shown viable cell attachment and growth on cellulose scaffolds derived from applies (Modulevsky et al., 2014).

**FIGURE 7:**
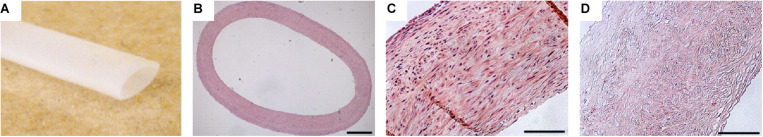
Example of engineered decellularized scaffold for vascular engineering (Quint et al., 2011). Scaffolds were produced by seeding smooth muscle cells onto a poly(glycolic acid) mesh. The scaffolds were cultured until the cells produced a contiguous ECM throughout the mesh and the mesh was almost entirely degraded. At this point, the scaffolds were decellularized, resulting in a cell-derived ECM tubular scaffold. Ultimately, scaffolds were re-seeded with endothelial cells and implanted in a porcine model. **(A)** Image of decellularized tubular scaffold. **(B)** Hematoxylin and eosin histological staining of cross-section of scaffold. Hematoxylin and eosin staining of scaffold microstructure **(C)** before decellularization and **(D)** after decellularization. Scale bars are 500 μm. Images were reordered from multiple panels, and lettering has been relabeled for consistency as part of this review article (Quint et al., 2011).

Decellularization has multiple advantages. First, the scaffold will inherently consist of a highly biocompatible material, which can assist in tissue production and maturation of the scaffold, with studies showing that similar cellular populations can be effectively re-seeded onto decellularized scaffolds. Typically, these scaffolds are also of sufficient mechanical strength for the specified application, given their origin. The scaffolds will also likely contain matrix-bound growth factors and other biochemical cues that can influence cell behavior upon re-seeding (Zhou et al., 2020). Some studies have also tissue engineered cell sheets, decellularized these sheets, and then used them for re-seeding with a new cellular population (L’Heureux et al., 1998; Quint et al., 2011). This approach can produce entirely biologically derived scaffolds (Figure 7), with the particular advantage of potential usage of only autologous cells and components. Decellularization has been used jointly with many other methods for tubular scaffold production. One study wrapped a cell-seeded, electrospun scaffold around decellularized aorta fitted with a cast polymeric stent as mechanical support for tracheal tissue engineering application (Ghorbani et al., 2017).

Decellularized tissue will also usually maintain its native structure to some extent (Totonelli et al., 2012), which can be useful. However, the resultant properties of the decellularized tissue will likely be altered during the decellularization process (Partington et al., 2013). Decellularized tissues need to undergo significant characterization to ensure that the resulting scaffold can still be used in the desired application. Many studies have utilized decellularized tissues originating in regions differing from the ultimate region of application, i.e., use of decellularized amniotic membrane as a vascular scaffold (Lee et al., 2012). While this strategy may be very effective, the presence of matrix-bound growth factors that are not associated with the desired tissue can affect the behavior of the newly seeded cells. A major disadvantage to the use of decellularized scaffolds is sourcing the tissue for decellularization. Typically, researchers use xenografts, which can potentially initiate negative responses depending on the origin of the scaffold and re-seeded cells. Secondly, the use of tissue derived from organisms can induce high variability between scaffolds. However, with proper quality control and analysis, decellularized scaffolds are a powerful tool in the tissue engineering of tubular systems.

### Other Methods

Various other methods exist for modeling tubular systems. Most notable among these is the use of microfluidic systems. Microfluidic systems typically utilize 2D cell culture, which is compatible with epithelial cultures. As such, various advancements in our understanding of cellular mechanisms have been developed through microfluidics. These structures are generally not tubular or 3D, and we, therefore, have not included them in our review of available manufacturing mechanisms for tissue engineered scaffolds. However, microfluidic technologies for epithelial engineering have been extensively reviewed elsewhere (Wong et al., 2012; Ahadian et al., 2018).

## State-Of-The-Art for Tubular Tissue Engineering

Here, we discuss specific applications for the manufacturing techniques discussed above. As mentioned in the introduction, we will focus on tissue engineered vasculature, intestine, and trachea, particularly considering systems that have applications in drug testing and discovery.

### Vascular Systems

Vasculature is one of the most commonly tissue engineered structures in the body. Various reviews have been specifically written about tissue engineering vasculature (Nerem and Seliktar, 2001; Stegemann et al., 2007; Song et al., 2018), with reviews even written specifically about using electrospinning for vascular tissue engineering (Rocco et al., 2014). Here, we seek to highlight some of the innovations in vascular tissue engineering from a tubular scaffold manufacturing standpoint in addition to discussing recent approaches.

Many tissue engineered vascular models rely on a population of smooth muscle cells to produce the relevant ECM on the scaffold support (Seliktar et al., 2003; McFetridge et al., 2007; Lee et al., 2007; Nieponice et al., 2008; Zhang et al., 2013; Strobel et al., 2018a). However, some systems have also used fibroblasts, mesenchymal stem cells, or other stem-like progenitors for this purpose (Shin’oka et al., 2005; Vaz et al., 2005; Wang et al., 2009; Rayatpisheh et al., 2014; Bertanha et al., 2014; Jung et al., 2015; Gui et al., 2016; Rabionet et al., 2018; Strobel et al., 2018b). Much of the literature regarding vascular design is targeted at the ultimate use of the structure as an implant or vascular graft. This objective allows for the endothelialization of the structure *in vivo*. However, for benchtop models, endothelial cells must also be included. Some models have utilized only endothelial cells (Matsuda, 2004; Lovett et al., 2007; Xi-Xun et al., 2008; Zhang et al., 2013; Wang et al., 2014; Zhou et al., 2016; Zhao et al., 2018), but many of the more complex models involve a co-culture of endothelial cells with an ECM-depositing cell type (Niklason and Langer, 1997; L’Heureux et al., 1998; Niklason et al., 1999; Boland et al., 2004; Opitz et al., 2004; Swartz et al., 2005; L’Heureux et al., 2006; Lovett et al., 2008; Yang et al., 2009; Neff et al., 2011; Han et al., 2011; Cheng et al., 2017; Ju et al., 2017; Atchison et al., 2017; Cui et al., 2019; Atchison et al., 2020).

Given the layered structure of native blood vessels (Figure 1A), many models utilize an approach where the ECM-forming cell type is first seeded, followed by a secondary seeding step of endothelial cells. This seeding method is compatible with virtually any tubular scaffold construction. An earlier model for tissue engineered vasculature used a series of three layers to mimic native vascular structure. The basis for the scaffold was formed through a fibroblast-derived cell sheet that was rolled into a multi-layered tube. The layers fused together in culture before subsequent dehydration, resulting in decellularization, for further seeding. This support tubing was wrapped with a second, living sheet of fibroblasts followed by injection of endothelial cells into the lumen, at which point the vessel was subjected to fluid shear (L’Heureux et al., 2006). This model was not used as a benchtop system but was ultimately implanted into humans (L’Heureux et al., 2007), significantly driving the field of vascular tissue engineering forward. However, this technique, while generally successful, took an extended period of culture (∼28 weeks) to produce the finalized vessel.

Various early studies solidified the usage of stratified cell layers in vascular tissue engineering, particularly with regard to different tubular scaffold construction methodologies. Some studies focused on the initial seeding of the scaffold with smooth muscle cells or fibroblasts (Boland et al., 2004; Yang et al., 2009; Neff et al., 2011). Other studies used cell-compatible injection molding techniques to create a tissue-like structure as the scaffold (Swartz et al., 2005; Liu et al., 2007). Some of these studies were able to demonstrate response of smooth muscle cells to vasoactive reagents, indicating the potential of these constructs for drug testing (Swartz et al., 2005; Liu et al., 2007). However, these particular studies produced structures that lacked an endothelium for these tests (Swartz et al., 2005; Liu et al., 2007), limiting similarity to native tissue. A more recent study used a rolling technique to produce the medial/adventitial regions of the blood vessel (Jung et al., 2015). Mesenchymal stem cells were used to produce cell sheets, which were rolled into a tube with four concentric layers. These sheets fused together during culture in a bioreactor. The lumen was then seeded with a population of endothelial progenitor cells and cultured under flow conditions. Exposure of the vessels to a vasoconstrictor, phenylephrine, elicited a constrictive response by the cells. The vessels also dilated under increased flow. The study also examined the adhesion of monocyte-like cells under exposure of the vessel to TNF-α, an inflammatory cytokine. TNF-α can upregulate production of adhesion molecules on vascular walls to aid in leukocyte binding. Increased binding was observed for this system with exposure to TNF-α. However, the researchers did not analyze their structures for the formation of endothelial junctions (Jung et al., 2015).

Another study focused on the development of a bioink and a co-axial extrusion printer system to enable the direct printing of tubular structures containing cells (Cui et al., 2019). In this setup, the walls of the resultant vessel were constructed of crosslinked gelatin methacrylate, containing smooth muscle cells. The lumen was formed by extruding Pluronic F127, a bioinert polymer surfactant hydrogel which can be dissolved under certain conditions, containing endothelial cells. The structure was allowed to set before the Pluronic F127 layer was removed from the lumen. During the removal process, some of the endothelial cells adhered to the interior walls of the lumen, resulting in a gelatin methacrylate layer containing smooth muscle cells with an interior lining of endothelial cells, remnant after the lumen clearance. Vascular permeability was assessed on similar structures possessing a non-tubular geometry, finding that the endothelial layer showed decreased permeability. The structures were also subjected to a vasodilator, acetylcholine, and showed dilation upon exposure (Cui et al., 2019). A major benefit to structures produced in this manner, or similar manners, is the vast array of feasible shapes and channels that can be produced through 3D printing. Studies are increasingly focusing on the development of microvasculature (Wang et al., 2014; Abaci et al., 2016; Lei et al., 2019), which can provide more realistic environments in other models, such as the intestine.

Various models have also been produced to mimic and study specific disease states. Tissue engineered vasculature was developed to model Hutchison-Gilford Progeria Syndrome (HGPS), which is associated with increased prevalence of cardiovascular disease and is associated with dysfunctional smooth muscle cells in vasculature (Atchison et al., 2017, 2020). This model was developed using induced pluripotent stem cells (iPSCs) and endothelial progenitor cells. The iPSCs were derived from healthy fibroblasts or fibroblasts an HGPS mutation, then differentiated toward smooth muscle cells. Tissue engineered vasculature was constructed by injecting collagen, containing smooth muscle cells, into a mold, following by gelation of the cell-seeded collagen. Endothelial progenitors were then perfused into the lumen for seeding (Atchison et al., 2017, 2020). The authors were able to observe differences in vasoactivity between healthy and diseased cells, as monitored by examining vasodilation and vasoconstriction using acetylcholine and phenylephrine, respectively (Atchison et al., 2020). This study and others highlight the major potential for use of tissue engineered vasculature as a model for drug testing/discovery.

### Intestinal Systems

Intestinal tissue engineering represents a particularly challenging field in the difficulty of producing a functional intestinal epithelium, given the numerous types of epithelial cells present in the native intestine. Further, the intestine has a complex 3D cross-section of villi and crypts, which also relates to the cellular distribution in the intestine (Santos et al., 2018). To reconstitute these structures, numerous studies have developed intestinal models using microfluidics or 2D cell culture substrates. However, these models do not fully mimic the complex native environment of the intestine (Bein et al., 2018). As such, 3D tissue engineered models have also been developed, which allow for the recapitulation of some of the aspects of the native intestine.

Some of the earlier tissue engineered models of the intestine were developed by rolling polymeric tubes and seeding these scaffolds with organoids derived from native murine colons. These structures were successfully transplanted into rats (Grikscheit et al., 2002). These structures were also used to treat induced short bowel syndrome in rats. The tissue engineered structures were beneficial for survival and gut function versus sham controls (Grikscheit et al., 2004). These studies indicated the possibility for the development of further tissue engineered benchtop models.

One model developed non-tubular 3D villus and crypt geometries using casting techniques (Yu et al., 2012). Scaffold construction was performed by casting collagen onto a negative of the villus-crypt structure. These structures were seeded with an enterocyte-like cell line (Caco-2 cells) and analyzed versus a flat system, i.e., without villi. The permeability of the cellularized structures was gauged using electrical measurements I and permeation by two drugs, finding higher permeability coefficients in the 3D structures (Yu et al., 2012). This experimental design has since been used with various other materials, mainly porous polymeric structures, to monitor the effects of bacterial culture with enterocytes (Costello et al., 2014; Ladd et al., 2019) and has recently been formed into tubular structures for implantation (Ladd et al., 2018).

Perhaps one of the most complete tissue engineered tubular models was developed for the intestine, using a cellularized, stratified silk scaffold (Chen et al., 2015) with a bioreactor, designed to simulate gut motility (Zhou et al., 2018). A casting approach was used to generate tubular silk scaffolds (Figure 3). Primary intestinal myofibroblasts were seeded within collagen into the silk scaffold and the interior lumen was subsequently lined with a co-culture of an enterocyte-like cell line (Caco-2) and goblet-like cell line (HT29-MTX) by luminal injection. The resultant cultures showed mucus production and tight junctional formation throughout the epithelial cell layer. Further, an intraluminal oxygen gradient was detected (Chen et al., 2015). Iterations on this model have also included colonoid-derived epithelium (Chen et al., 2017) with monocyte cultures and examination for macrophage infiltration through the epithelium (Roh et al., 2019). A bioreactor was also developed and applied to this system to provide pulsatile, peristaltic-like stimulation to these constructs (Zhou et al., 2018).

Many other non-tubular systems have also successfully modeled various aspects of the gastrointestinal tract. For example, an extensive bioreactor system was developed to simulate the microbial populations of the intestine (Van den Abbeele et al., 2012). A recently developed microfluidic model was also able to recapitulate the intestinal epithelium and associated microbial population with an adjacent vascular structure (Jalili-Firoozinezhad et al., 2019). This design resulted in the formation of a villus-crypt structure with polarized epithelial cells. This device also included sensors for monitoring oxygen content non-invasively, allowing for monitoring of oxygen gradients between the intestinal lumen and adjacent vascular lumen chambers (Jalili-Firoozinezhad et al., 2019). These types of devices are particularly useful as basic science platforms and can help inform the design of larger tubular systems. Currently, many of the tissue engineered intestinal models are focusing on the examination of the gut microbiome or immune components. As these models are developed further and our understanding of some of these aspects of intestinal biology improves, focus will likely shift to the testing of drugs in these tissue engineered systems.

### Tracheal Systems

Unlike the *in vitro* models discussed for vasculature and the intestine, the outlook for tracheal tissue engineering to date largely concerns implantable scaffolds for tracheal replacement. In recent years, research has been targeted toward improving *in vitro* preparation methods such as optimizing decellularization through enzymatic, detergent (Zhong et al., 2019), vacuum-assisted (Butler et al., 2017), and chemical-based techniques (Batioglu-Karaaltin et al., 2019). For example, enhanced enzymatic approaches have drastically reduced tracheal decellularization time (Giraldo-Gomez et al., 2019; Wang et al., 2020). Importantly, the reduced preparation time had no adverse effects on tracheal ECM structure or biomechanical properties and evaded immunogenic or inflammatory responses when implanted *in vivo.* This result highlights the promise of advanced decellularization methods for production of tracheal implants. Despite success in these areas, decellularization methods require availability of human tracheal donors, and decellularized scaffolds can possess altered mechanical properties compared to native (Partington et al., 2013).

A major requirement for tracheal tissue engineering is sufficient mechanical support to resist pressurization. As such, many tissue engineered tracheal models have been created through combination of different manufacturing methods, incorporating a mechanical support structure with a cell-seedable scaffold. Tissue engineered trachea have been produced using a combination of 3D printing and decellularization methodologies (Johnson et al., 2016). This approach offers many advantages, including improved structural and mechanical support versus decellularized tissue alone, while maintaining biocompatibility and a native-like ECM. These hybrid 3D printed-decellularized scaffolds showed comparable resistance to compression versus native tissue and higher resistance to compression versus a decellularized scaffold alone (Johnson et al., 2016). Scaffolds produced through combination of 3D printing and electrospinning also possessed sufficient mechanics (rotation angle, elastic modulus, elongation ratio, and tensile strength) for recapitulation of the trachea, while still demonstrating a high degree of cellular attachment (Ahn et al., 2019). Other studies have mimicked native tracheal structure by incorporating C-shaped rings onto 3D printed tubular designs, thusly producing similar mechanical profiles to *in vivo* (Ott et al., 2016; Best et al., 2018).

Other studies have looked to characterize biocompatibility and regenerative capabilities of tracheal grafts via transplantation into rabbit models. For example, 3D solvent-based casting techniques have been utilized to fabricate tubular scaffolds, which demonstrate vascularization and differentiation of ciliated, mucus producing tracheal epithelium 4 weeks post-implantation (Park et al., 2015). Similarly, 3D bioprinting of a tubular tracheal tissue, containing layers of PCL and autologous epithelial cells and chondrocytes (Figure 6), demonstrated complete regeneration of respiratory epithelium and long-term stability of tracheal function (Park et al., 2019). However, further studies are needed to assess possibility of adverse immune or inflammatory processes and to promote chondrocyte regeneration of cartilage for mechanical support. Additionally, sufficient luminal airflow and gas tightness is necessary (Hsieh et al., 2018) and fundamental to the success of tracheal implants when positioned at the native air-liquid interface.

In summary, many studies have shown the successful fabrication of layered, flexible and structurally supportive tubular tracheal models, with characterization and comparisons made *in vitro* using techniques such as high-resolution microscopy, immunofluorescence, histological staining, and mechanical testing. Although some studies have looked to address microbial properties and response to pathogen invasion (Kang et al., 2019), to date, most tissue engineered tracheal model systems focus on developing implantable scaffolds. The limited examples of models which address toxicology or drug testing may be associated with the difficulty of established a 3D air-liquid interface *in vitro*. Further, the use of high throughput microfluidic and 2D systems for drug stimulation and discovery is well-established in this field (Ahadian et al., 2018).

## Future Perspectives

The field of tissue engineering for tubular systems has undergone various innovative steps over the course of its history. However, tissue engineered models still do not fully resemble native tissue. This lack of resemblance is partially due to the numerous cell types that require representation. For example, few systems include neural and immune components, which are increasingly connected to tissue function and homeostasis (Veiga-Fernandes and Artis, 2018). Further, in intestinal tissue engineering, numerous cell types are necessary for making only the epithelium, which can drastically complicate culture setup. Further innovations in scaffold manufacturing have the potential to solve these issues. Bioprinting can potentially place various cell types in a stratified manner, increasing the feasibility of producing cellularly complex systems. However, the cost of producing or purchasing a bioprinter capable of constructing such stratified structures is currently too high to be widely available. As such, innovations in the surrounding technologies, i.e., multi-nozzle systems, integrated culture capabilities, etc., can significantly increase feasibility. Other techniques involving tubular self-assembly can accomplish some of these tasks at lower cost. Researchers should also consider the production of modular systems for improving model complexity. Designing scaffolds that can be placed into a co-culture upon reaching maturity allows for a wider range of culture conditions and experiments without necessarily requiring expensive fabrication equipment.

Other factors complicating current efforts in tubular tissue engineering involve the application of native-like conditions to the resultant scaffolds. For example, while some studies have developed native-like culture conditions using bioreactors (Zhou et al., 2018), most studies rely on media conditions to produce tissue models. Many of the cells present in these systems are mechanoresponsive, as evidenced by the dynamic physiological conditions *in vivo*, indicating that further stimuli must be applied to reach native-like conditions *in vitro*. For this particular limitation, study designs must consider compatibility of the scaffold with a subsequent bioreactor system. Scaffolds can potentially be fabricated inside a bioreactor using many of the techniques above, such casting and printing. Alternatively scaffolds can be produced with dynamic material components, like piezoelectric materials, that can potentially provide mechanical stimuli to a scaffold using an inherent material property.

A final piece to consider for improving tubular scaffold efficacy is engineering of scaffold walls to generate multiple cellular microenvironments within a singular scaffold. For example, scaffolds can be produced to support formation of microvasculature (Chang and Niklason, 2017). Use of such a scaffold in an intestinal tissue engineering application can provide cells with nutrients through microvasculature channels, which can theoretically simplify bulk fluidic bioreactor designs. Similar concepts can be applied to tracheal tissue engineering, in designing scaffolds that can support cartilage formation, while simultaneously providing a face for epithelial formation. Many of the scaffold manufacturing techniques presented in this review have the potential for microstructural engineering within the scaffold bulk, and consideration of desired microenvironments when designing a fabrication process can have considerable benefits in the final results.

As tissue engineered tubular systems progress toward functional tissues, these systems can be used for drug testing and discovery. Many systems have already begun to show active responses to different drugs (Jung et al., 2015; Atchison et al., 2017, 2020). However, a major factor that is still lacking is the ability to monitor these systems in real-time. Most tissue engineered systems, particularly those with 3D structural features, require endpoint analysis. Studies typically use analytical techniques like immunostaining or blotting to determine the cellular response and activity in the tissue engineered system. While these techniques are very useful and informative, they can also result in a lengthened study period, in that they will often require titration or larger sample numbers to reach sufficient statistical power for analysis.

Some studies have begun to integrate non-invasive techniques, like electrical measurements, that can be performed during culture. These techniques provide real-time data on the tissue engineered system that can be compared to typical endpoint analyses. For example, a common method for assessing epithelial barrier formation, with reference to drug or toxicology studies, is transepithelial electrical resistance (TEER) (Benson et al., 2013; Srinivasan et al., 2015). However, TEER apparatus are somewhat limited due to the rigid structure of electrode probes, which fails to conform to the complex architecture of advanced tissue engineered models. Other common means for monitoring tissue engineered tubular models include various material analyses, such as mechanical analysis, or biological analytical methods, such as histology and immunostaining. However, these methods are also more difficult to apply in tubular scaffolds due to their geometry, further complicating assessment in these tissue engineered systems. In answer to these limitations, some innovative solutions have been demonstrated including the use of an electroactive polymer scaffold, which can monitor real-time cell adhesion, growth, and migration during culture (Pitsalidis et al., 2018), with potential for a tubular setup for tissue engineering. Additionally, electrodes have been fabricated in organ-on-chip devices (Henry et al., 2017), microfluidics (Curto et al., 2017), and flexible polymer substrates (Ferro et al., 2019; Kalmykov et al., 2019) to monitor barrier integrity in complex cell cultures. These examples highlight the potential of flexible and microelectronic fabrication methods in monitoring complete barrier formation in future 3D tissue-engineered models. However, while measurements like TEER, are very common in 2D systems, these measurements have not yet been widely adapted to larger-scale 3D systems.

Overall, we have discussed the various mechanisms by which tubular scaffolds can be constructed for tissue engineering. We divided the available manufacturing methodologies into five major categories: casting, electrospinning, rolling, 3D printing, and decellularization. Innovations for every one of these methodologies are still being generated today, with continuous new advancements in fabrication of scaffolds and tissue engineered systems. Methods like 3D printing and self-assembled rolling scaffolds allow for simultaneous advancements in ease of manufacturing and system complexity, driving toward tissue engineered systems that truly mimic native tissues. As these systems are developed, we will soon see their viable use in testing drug safety and efficacy in future biomedical studies.

## Author Contributions

AB, DT, and RO were responsible for design of the contents of the article, including the subject and general outline. AB and SB were responsible for the majority of the writing of the article. DT assisted in writing initial drafts of the article. SB and DT designed and produced the custom figures for the article. RO provided feedback and guidance for construction of the article. All authors have read the article, provided feedback, and given approval.

## Conflict of Interest

The authors declare that the research was conducted in the absence of any commercial or financial relationships that could be construed as a potential conflict of interest.
